# The differential extension in dsDNA bound to Rad51 filaments may play important roles in homology recognition and strand exchange

**DOI:** 10.1093/nar/gkt867

**Published:** 2013-09-30

**Authors:** Claudia Danilowicz, Alexandra Peacock-Villada, Julea Vlassakis, Adrien Facon, Efraim Feinstein, Nancy Kleckner, Mara Prentiss

**Affiliations:** ^1^Department of Physics and ^2^Department of Molecular and Cellular Biology, Harvard University, Cambridge, MA 02138, USA

## Abstract

RecA and Rad51 proteins play an important role in DNA repair and homologous recombination. For RecA, X-ray structure information and single molecule force experiments have indicated that the differential extension between the complementary strand and its Watson–Crick pairing partners promotes the rapid unbinding of non-homologous dsDNA and drives strand exchange forward for homologous dsDNA. In this work we find that both effects are also present in Rad51 protein. In particular, pulling on the opposite termini (3′ and 5′) of one of the two DNA strands in a dsDNA molecule allows dsDNA to extend along non-homologous Rad51-ssDNA filaments and remain stably bound in the extended state, but pulling on the 3′5′ ends of the complementary strand reduces the strand-exchange rate for homologous filaments. Thus, the results suggest that differential extension is also present in dsDNA bound to Rad51. The differential extension promotes rapid recognition by driving the swift unbinding of dsDNA from non-homologous Rad51-ssDNA filaments, while at the same time, reducing base pair tension due to the transfer of the Watson–Crick pairing of the complementary strand bases from the highly extended outgoing strand to the slightly less extended incoming strand, which drives strand exchange forward.

## INTRODUCTION

RecA family proteins play important roles in DNA recombination and repair (1). Two important members of this family are RecA and Rad51, which occur in bacteria and eukaryotes, respectively (2). At the beginning of a repair or recombination process, a region of single-stranded DNA (ssDNA) is formed at a double-stranded DNA (dsDNA) break (3,4). The single-stranded region, referred to as the incoming strand, is covered by a recombinase protein forming a helical filament. This filament searches a genome for sequence homology by rapidly binding and unbinding to dsDNA until homology is found. The two strands in the dsDNA are known as the outgoing and complementary strands. The initial binding of dsDNA to the recombinase-ssDNA filament is sequence independent. The binding does not become sequence dependent until homology is tested. Thus, early binding stages will be the same regardless of whether or not the dsDNA is homologous to the ssDNA. It is believed that during homology recognition, the bases in the complementary strand test for homology by base flipping which can transfer the Watson–Crick pairing of those bases from the outgoing strand to the incoming strand (5). If the sequence of the complementary strand is homologous to the sequence of the incoming strand, base flipping allows the complementary strand to Watson–Crick pair with the incoming strand, forming heteroduplex dsDNA bound to site I, which leaves the bases in the outgoing strand unpaired.

**Figure 1.**
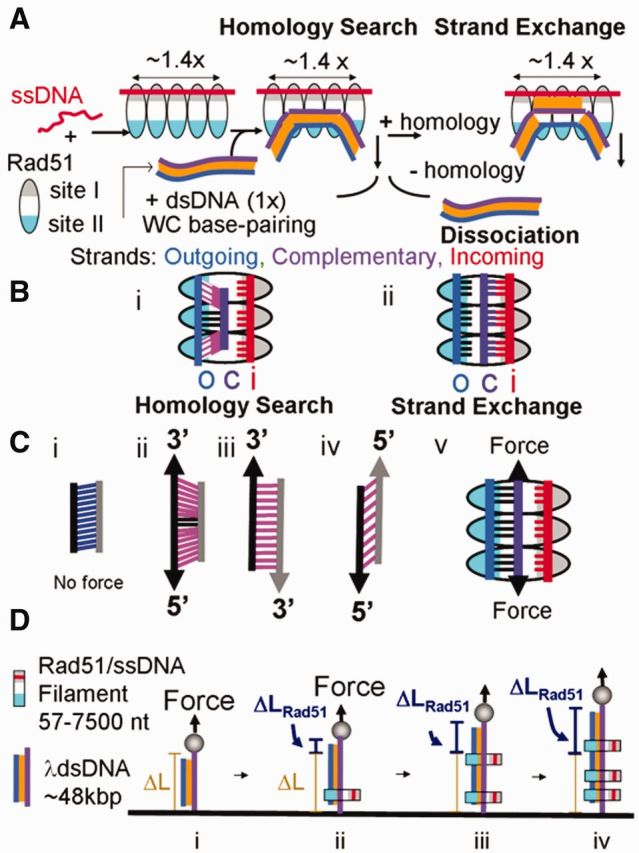
Rad51 strand exchange (**A**) Schematic representation showing incoming strand ssDNA (red line), outgoing strand (blue), complementary strand (purple), Watson–Crick pairing (orange), and Rad51: site I (gray region of oval) and site II (blue region of oval). (**B**) Extension and tension on dsDNA bound to Rad51-ssDNA filaments with pink highlighting base pairs under tension (i) dsDNA bound to the presynaptic filament (ii) dsDNA bound to a Rad51-ssDNA filament in the poststrand exchange state. (**C**) Extension and tension on dsDNA with the same color scheme used in (B) (i) in the absence of external force, (ii) with external force applied to the 3′5′ ends of one strand, (iii) force applied to 3′3′ opposite ends, (iv) force applied to 5′5′ ends and (v) with external force applied to the 3′5′ ends of the complementary strand in the homology searching complex (**D**) Assay for measuring the extension of dsDNA due to the binding of Rad51-ssDNA filaments (i) λ dsDNA tethered between a capillary tube and a magnetic bead under force (arrow) that is extended without [ΔL, (i)] and with [ΔL_Rad51_ (ii–iv)] binding of non-homologous Rad51-ssDNA filaments.

In order for strand exchange to occur through base flipping, the projections of the extension of the DNA along the direction of the helical axis must be similar for all three strands. In RecA-ssDNA filaments, bound dsDNA is extended such that the projection of the average extension along the direction of the helical filament is 1.5× the B-form extension, and the bound dsDNA is underwound (6). Similarly, dsDNA bound to Rad51-ssDNA filaments is underwound and extended 1.5× the B-form length (7), as illustrated in Figure 1A.

In RecA, though the average DNA extension along the direction of the helical axis is 1.5× the B-form length, the spacing of the bases is not uniform (8). The bases consist of nearly B-form triplets separated by large rises. In RecA, experimental results suggest that the incoming strand is bound to site I in the protein, which places the incoming strand near the center of the helical RecA-ssDNA filament (8). In contrast, the known structure of DNA bound to RecA suggests that the outgoing strand is bound to residues that are much more distant from the center of the helical filament than the incoming strand (9). Thus, when dsDNA binds to a RecA-ssDNA filament the outgoing strand backbone in the dsDNA is more extended than the incoming strand backbone despite their similar projections along the axis of the filament. Furthermore, when dsDNA is bound to RecA-ssDNA filaments both the incoming strand and the outgoing strand are believed to be bound directly to the protein whereas the complementary strand is believed to be bound dominantly via Watson–Crick pairing with either the outgoing (9) or the incoming (8,9) strand.

The rises between the triplets in the incoming and outgoing strand are maintained by DNA protein contacts, but the rises in the complementary strand are maintained dominantly by the force exerted by the paired bases that connect to the unsupported complementary strand (8,9), though interactions with the L1 and L2 loops may provide some mechanical support. In RecA, the large extensions in the rises between the triplets in the incoming and outgoing strands result in mechanical tension on the base pairs that connect the complementary strand to its Watson–Crick pairing partners. The tension is greatest in the initial homology searching state where the complementary strand is Watson–Crick paired with the outgoing strand. The tension is largest in this state because the outgoing strand has an extension that is significantly larger than the extension of the incoming strand (9), as illustrated in Figure 1Bi.

The large stress on the base pairs is believed to limit the number of base pairs that can bind to the RecA-ssDNA filament unless the system undergoes strand exchange or an external force provides mechanical support that reduces the internal tension in the bound dsDNA (10). In the absence of an applied external force this tension results in a checkpoint that limits the initial sequence independent binding to ∼9 bp (11). This checkpoint also promotes the rapid unbinding of non-homologous dsDNA. The existence of the checkpoint allows the RecA system to overcome the speed-stability paradox that would otherwise require the system to tradeoff sequence stringency against searching speed (12).

The differential extension of the complementary and outgoing strands not only limits the number of triplets that can be bound in the initial sequence independent state, but it also drives strand exchange forward in cases where the bound dsDNA is homologous to the ssDNA in the RecA-ssDNA filament. Strand exchange is driven forward by the decrease in base pair stress that occurs when the pairing of the complementary strand bases is transferred from the very highly extended outgoing strand to the slightly less highly extended incoming strand, as illustrated in Figure 1Bii. If nine contiguous homologous base pairs make the transition to the intermediate strand-exchanged state, the tension on the dsDNA is sufficiently reduced to allow another triplet to bind to the RecA-ssDNA filament in the sequence independent initial state. If that triplet is homologous, strand exchange will again be driven forward by a decrease in base pair tension.

Rad51 is a member of the RecA family of proteins that facilitates homology search during strand exchange to repair double-stranded breaks (DSBs) and during homologous recombination in meiosis (7,13,14). Given that both RecA and Rad51 extend and underwind bound dsDNA, it is possible that Rad51 also uses differential extension of the bound dsDNA strands to promote the rapid binding of non-homologous filaments as well as driving strand exchange forward for homologous filaments. It is known, however, that there are significant structural and functional differences between RecA and Rad51 (15,16). Considering both the similarities and the differences between these two proteins, it is important to test whether or not differential extension of dsDNA bound to Rad51-ssDNA filaments plays significant roles in homology recognition and strand exchange.

In the following, we perform such experiments. In particular, we study the binding of non-homologous Rad51-ssDNA filaments to dsDNA being pulled from the 3′5′ ends and compare the results with those obtained when the dsDNA is pulled from the 3′3′ and 5′5′ ends. We used our magnetic tweezers apparatus to apply forces to the ends of 3′5′, 3′3′ and 5′5′ dsDNA in the presence of non-homologous Rad51-ssDNA filaments, as represented in Figure 1. *In vivo*, non-homologous dsDNA must rapidly unbind from Rad51-ssDNA filaments; however, consistent with earlier results obtained with RecA-ssDNA filaments (10), experimental results show that applying a force >54 pN on the 3′5′ ends of the dsDNA allows non-homologous dsDNA to be extended by binding to Rad51-ssDNA filaments, where the stable binding is maintained as long as the force is applied. If the force is removed, the non-homologous dsDNA rapidly unbinds from the filament, suggesting that the applied force is required to maintain the stable binding of the non-homologous dsDNA.

In order to maintain the B-form structure of the dsDNA we work below the overstretching force of ∼65 pN, where dsDNA is known to extend to ∼1.7 times its B-form length for all pulling techniques. We performed similar experiments with homologous filaments, where the force was applied after the filaments had begun to undergo strand exchange. The results suggest that Rad51 does use differential extension to promote the unbinding of non-homologous dsDNA and to drive the strand exchange of homologous dsDNA, just as RecA does; however, the differences between the results for RecA and for Rad51 hint at structural differences between the two systems.

## MATERIALS AND METHODS

### Applying force along a dsDNA molecule

Experiments were carried out in a square microcell (0.8 mm× 0.8 mm) containing a round inner capillary (0.55 mm in diameter) closed at its ends. The inner capillary was modified by adsorption of 1 mg/ml extravidin in phosphate-buffered saline pH 7.4 overnight. Double-stranded linear λ genomes (New England Biolabs) were modified by addition of biotinylated oligonucleotides yielding 3′5′, 3′3′ and 5′5′ dsDNA constructs (10). Samples were prepared by hybridizing and ligating the complementary oligonucleotides. Two, three and four reaction steps were required for the 3′3′, 3′5′ and 5′5′ samples, respectively. Ligation steps were done in the presence of a thermostable DNA Ligase (Ampligase, Epicentre, Madison, WI, USA). The oligonucleotides at both ends of all these structures included a ssDNA tail [(dT)_7_-(biotin-dT)_6_] to allow free rotation of the bonds in the phosphate backbone so that no steady state torque can be maintained in the dsDNA molecules. After each modification step was completed, the dsDNA samples were washed three times using Amicon YM-100 filters (Millipore, USA) and 70 mM Tris buffer pH 7.6. The final concentration was determined by the absorbance at 260 nm and the dsDNA samples were diluted to 7 µg/ml before use. These molecules were tethered to capillary walls at one end and to superparamagnetic beads (4.5 µm in diameter, 4 × 10^8^ beads/ml, Invitrogen) at the other end. A 0.2 µl aliquot of each dsDNA in Rad51 buffer (70 mM Tris–HCl, 2 mM MgCl_2_, 1 mM dithiothreitol and 50 mM NaCl pH 7.6) containing 1 mM ATP (or ATPγS) was mixed with free human Rad51 (Abcam) (final concentration 10 nM) or with 0.5 µl aliquots of Rad51-ssDNA filaments and 5 µl of the suspension containing the beads. The resulting mixture containing dsDNA, free Rad51 or Rad51-ssDNA filaments, and beads was introduced into the microcell. The Rad51-ssDNA filaments were initially prepared by mixing 3 µM ssDNA (in nucleotides) from PCR fragments obtained using pcDNA3 as template [or M13mp18 phage ssDNA (New England Biolabs)], 1 µM Rad51 and 1 mM ATPγS in Rad51 buffer at 37°C for 30 min. The strand-exchange experiments were performed using ssDNA homolog to lambda dsDNA and 1000 or 5000 nt in length. The fragments were obtained by PCR from one of the ends of lambda phage and converted into ssDNA using lambda exonuclease. Purification was achieved using a Qiagen kit for ssDNA. Strand-exchange reactions were followed in 70 mM Tris–HCl, 2 mM CaCl_2_, 1 mM dithiothreitol and 50 mM NaCl pH 7.6 containing 2 mM ATP for increased activity (17). After an initial 2-min incubation at 37°C, the DNA molecules became tethered between the glass capillary surface and the extravidin-coated beads. The microcell was then placed in the magnetic tweezers apparatus (18) consisting of a stack of permanent magnets whose position relative to the cell can be varied to exert forces between 2 and 200 pN. The position of each bead at constant applied force was followed in real time at room temperature using an inverted microscope and a digital camera. Each bead was tracked by bead-tracking software and recorded using Matlab (www.mathworks.com).

Additional *in vitro* strand-exchange experiments were performed to evaluate our current protocol (Supplementary Methods, Supplementary Results and Supplementary Figure S1).

For the magnetic tweezers set up used in these experiments (18), the calibration of the relationship between the force on a magnetic bead and the position of the magnet was originally established using measurements of the Stokes drag on individual magnetic beads in glycerol. This calibration was checked using a Gauss meter to measure the magnetic field as a function of distance from the magnet and using the known magnetization of the magnetic beads to calculate the force. The beads have some variation in magnetization, which means that different beads experience a slightly different force at a given magnet position. The variation in the force is found to be ∼5% for these beads. We used the overstretching transition as a standard to calibrate the magnetization for each bead. Earlier work has shown that the overstretching force is 65 ± 1 pN (9), as long as the molecule is not rotationally constrained. The overstretching force for all pulling techniques is the same to within ∼1 pN. Thus, the reported forces are accurate to within 3%.

### Data analysis

Data analysis was performed using scripts custom-written in Matlab as described in our previous work (18). We developed an automated algorithm to detect filament binding from changes in extension (bead position) versus time curves. To identify intervals of constant growth, we choose an initial starting point at time *t* = 0. Then we performed linear least-squares fits on all subintervals of the growth curve beginning at the starting point and lasting at least 2 s. We chose as ‘best fit’ the line with the minimum absolute value of the sum of its residuals per unit length multiplied by the sum of the auto-correlation of its residuals. Then we set the endpoint of this best fit line as the starting point to seek a new best fit, proceeding iteratively until the entire growth curve was exhausted. We allowed the algorithm to skip short regions (<1 s) of high noise at the transitions between intervals with different growth rates. Because the exact positions of transitions between intervals with different growth rates are uncertain, we allowed a 0.5-s overlap between successive intervals. We considered intervals beginning >20 s after a change in force.

## RESULTS

We applied force to the ends of lambda dsDNA (48 502 bp) constructs including 3′5′, 3′3′, 5′5′, as illustrated in Figure 1C. Given that the binding of dsDNA to Rad51-ssDNA filaments extends the average projection length of the dsDNA along the helical axis to ∼1.5× the B-form length (7), we monitor the binding of dsDNA to Rad51-ssDNA filaments by measuring the extension of the dsDNA as a function of time, as illustrated in Figure 1D. The dsDNA lengths are measured with ∼10 nm accuracy every 0.2 s. When the dsDNA is pulled from the 3′5′ ends, the bead-to-capillary distance corresponding to the dsDNA length increases beyond that seen without Rad51, indicating ternary complex formation (ΔL_Rad51_, Figure 1D).

**Figure 2.**
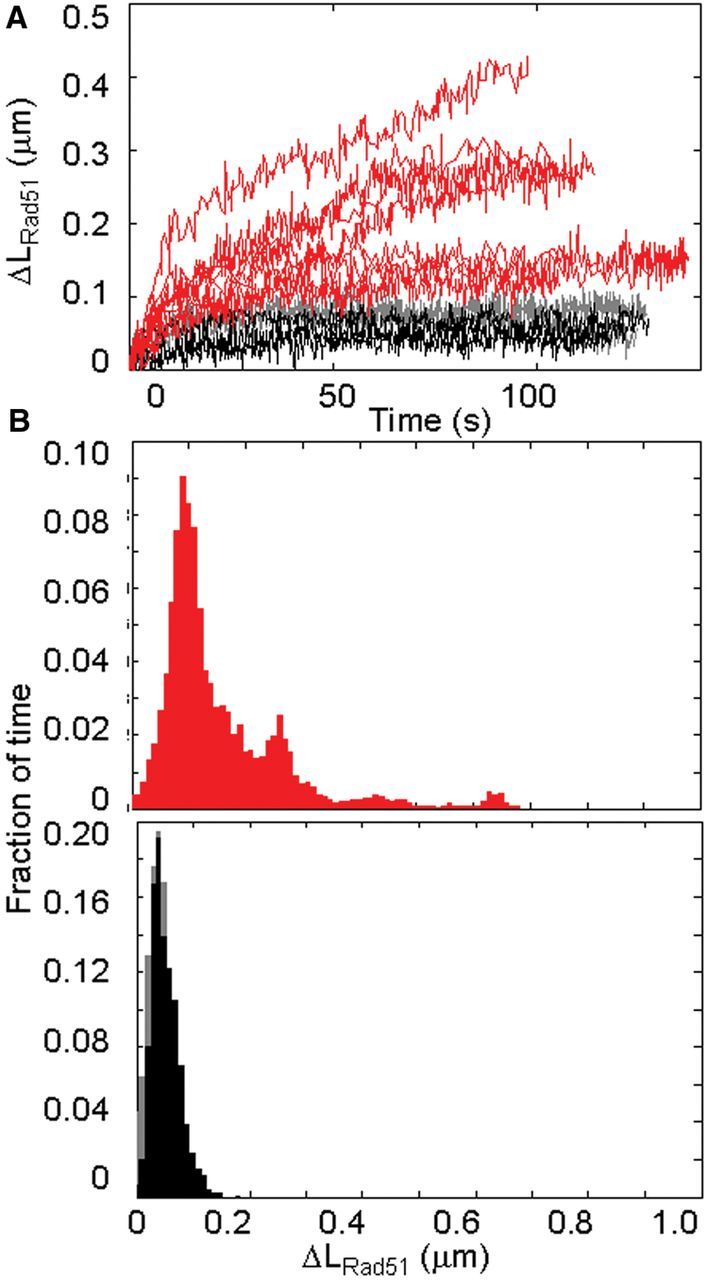
Binding of Rad51-ssDNA non-homologous filaments to dsDNA. (**A**) Extension versus time binding profiles for dsDNA pulled 3′5′ (red) in the presence of Rad51-ssDNA filaments 700 bases in length and negative controls with 10 nM Rad51 (black) and ssDNA and no protein (gray); force range 54–58 pN. (**B**) Frequency of extensions after 120 s of constant force for forces between 54 and 58 pN in the presence of Rad51-ssDNA filaments. (**C**) Frequency of extensions or the controls after 120 s of constant force for forces between 54 and 58 pN in the presence of 10 nM Rad51 (black) and ssDNA (gray). Filaments are 700-nt long obtained from pcDNA3 non-homolog to lambda phage dsDNA.

Binding signals were measured for single dsDNA molecules interacting with Rad51-ssDNAs filaments. We performed experiments using linear ssDNA with the following lengths: 400, 700, 1000 and 1600 nt prepared using a lambda exonuclease reaction (Supplementary Figure S2). We also performed experiments with circular ssDNA from M13mp18 phage (Supplementary Figure S3). Representative single molecule binding profiles for a 700-nt filament are shown in Figure 2. For these curves, the filaments were prepared and measured in a buffer containing ATPγS.

In the presence of non-homologous Rad51-ssDNA filaments of 700 bases in length, we observe an increase in extension of 3′5′ dsDNA held at constant forces between 50 and 60 pN. Figure 2 shows typical extension versus time curves for 3′5′ pulling between 54 and 58 pN. At these applied forces, the length of the dsDNA in the absence of filaments is still approximately the B-form length as shown by the gray and black curves (controls). At ∼65 pN dsDNA undergoes an overstretching transition where the length of the dsDNA increases by a factor of ∼1.7×, and the width of the overstretching transition is ∼2 pN (19–21); therefore, we did not study effects of forces >58 pN. In order to obtain the curves shown in Figure 2, a constant force of 40 pN was applied for 120 s, then the force was rapidly increased to a particular chosen force for 120 s, after which the force was lowered back down to 40 pN. A constant force of 40 pN was then applied for 120 s, and the cycles were repeated for several force values. In every case the change in extension of a single molecule was measured. The compilation of data at forces ≤58 pN reveals a series of peaks corresponding to integer numbers of filament lengths with an extension of ∼0.12 nm/bp (Supplementary Figure S2).

We ran two types of control experiments. In one type of control the ssDNA was present, but the solution did not contain any Rad51 protein. In the other control, free Rad51 was present in the concentration characteristic of the filament experiments (10 nM), but no ssDNA was present. Both types of control experiments showed similar results. In Figure 2 the control curves are shown in black (Rad51 only) and control experiments with ssDNA only showed similar curves (gray) overlapping with the black curves. Though Rad51 can bind to dsDNA, at such low concentrations very little binding is observed in the absence of force (22) or at low forces (23), and we do not observe significant changes in extension even at higher forces. Controls for 3′3′ pulling and 5′5′ pulling in the presence of ssDNA only were similar, but controls for 3′5′ pulling showed slightly shorter extensions (see below). In general, some controls show slight increases of extension during the first 20 s, but at applied forces <58 pN, the length of the controls is almost unchanged during the time interval between 20 and 120 s.

**Figure 3.**
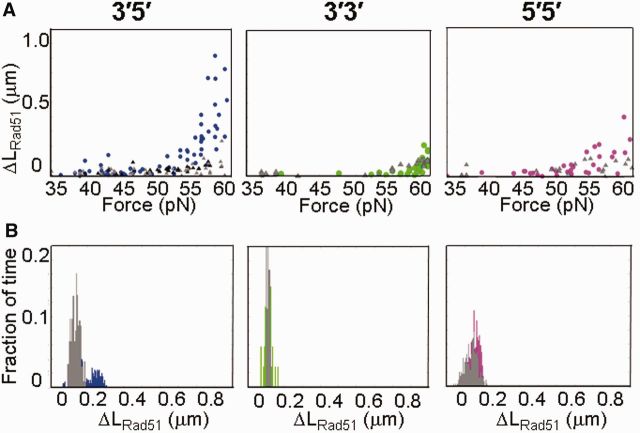
Comparison between different dsDNA pulling modes in the presence of non-homolog Rad51-ssDNA filaments. (**A**) Change in extension of lambda DNA pulled 3′5′ (blue), 3′3′ (green), and 5′5′ (magenta) after 120 s at constant force (over a range of forces between 35 and 60 pN). The control extensions for experiments with 1000-nt ssDNA fragments from pcDNA3 are shown in gray and controls in the presence of 10 nM Rad51 are shown in black. (**B**) Frequency of extensions after 120 s of constant force for forces between 50 and 53 pN; controls in the presence of ssDNA are shown in gray.

Changes in extension were measured in the presence of Rad51-ssDNA filaments by pulling from the 3′5′, 3′3′ and 5′5′ ends; the changes in extension for each case are shown in blue, green and magenta, respectively (Figure 3 and Supplementary Figure S4A). For forces >50 pN, molecules pulled from the 3′5′ ends show extensions that significantly exceed the extension of the controls.

The above results could imply that the force is required to overcome a barrier to binding, which allowed the system to reach a stable bound state that could be maintained in the absence of force. If this were true, once the dsDNA was bound to the filament, it would remain bound even if the force was subsequently removed. In order to test whether this was true, we decreased the force after the dsDNA had been extended by binding to the Rad51-ssDNA filament. Those experiments showed that reducing the force induced the dsDNA to unbind (Supplementary Figure S4B). Thus, the applied force is required to maintain the extension.

If the binding of a dsDNA to an ssDNA filament is sufficiently rare and the applied force is sufficient to allow the full extension of dsDNA along Rad51-ssDNA filaments, then the extension versus time curves should show pause points at extensions corresponding to full filaments lengths. This effect was observed when the analogous experiments were performed using RecA-ssDNA filaments (10). Similarly the extension/bp can be obtained by calculating the ratio of the extensions characteristic of the pause points to the number of nucleotides in the ssDNA. Histograms of the results are shown in Supplementary Figure S2. They show that the 0.12 ± 0.02 nm characteristic extension/nt is independent of nucleotide length, for lengths ranging from 400 to 1600 nt. Thus, the characteristic extension/nt is slightly smaller than the 0.17 nm/bp extension characteristic of RecA filaments. Given the experiments we have made, we cannot distinguish between two possible interpretations of these results: (i) The average projection of the extension along the filament axis is slightly smaller in Rad51 filaments than in RecA filaments and (ii) Rad51 does not completely cover the ssDNA, but the incomplete coverage is uniformly distributed over the filament for length ≥400 nt. If the effect were entirely due to incomplete ssDNA coverage, this would represent ∼70% Rad51 coverage.

In contrast with the results for 3′5′ pulling for forces <54 pN, pulling from the 3′3′ or 5′5′ ends results in extension increases that do not exceed the controls, as illustrated in Figure 3. Figure 3 shows the results for 3′5′, 3′3′ and 5′5′ pulling when non-homologous Rad51-ssDNA filaments are present in solution, and the black and gray symbols correspond to the controls. Figure 3A shows the measured extensions for the time interval between 105 and 120 s after the high constant force was applied as a function of the applied constant force. Figure 3A from left to right shows results for 3′5′ pulling, 3′3′ pulling and 5′5′ pulling where the colored symbols show results in the presence of the Rad51-ssDNA filaments, and the black and gray symbols show results for the controls with Rad51 only and ssDNA only, respectively. A comparison with corresponding results obtained using RecA is shown in Supplementary Figures S4 and S5.

Figures 3B shows histograms for the extensions averaged between 105 and 120 s for 50–53 pN. In the high force regime 3′5′ pulling shows extensions that exceed the controls by more than an order of magnitude, whereas 5′5′ pulling barely exceeds the controls and 3′3′ pulling actually shows extensions that are shorter than the controls.

**Figure 4.**
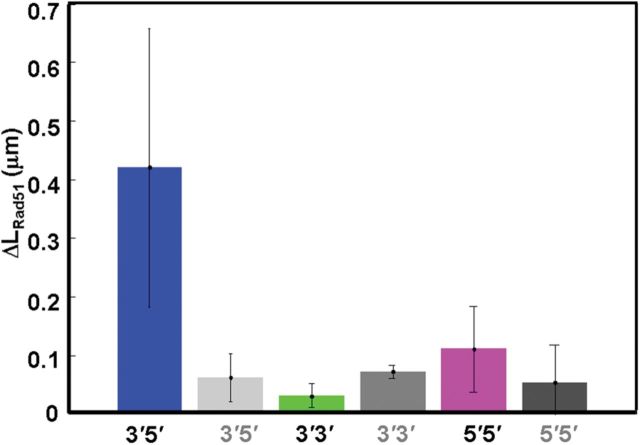
Bar graphs for ΔL_Rad51_ values between 56 and 58 pN after 120 s, and 1000 nt-filaments; λ dsDNA molecules are pulled 3′5′, 3′3′ and 5′5′ shown in blue, green and magenta, respectively, and controls are shown in gray. In these experiments 1000 nt- Rad51-ssDNA filaments from pcDNA3 were prepared in a buffer containing ATPγS and measured in a buffer containing ATPγS.

Figure 4 shows bar graphs of the average extension during the time interval between 105 and 120 s for forces between 56 and 58 pN, where the color code is the same as in Figure 3. The error bars indicate the standard deviation in the measured lengths. For 3′5′ pulling, the positives exceed the controls by many standard deviations. In contrast for 5′5′ pulling, the error bars for the positives and the controls overlap, and the 3′3′ results are slightly shorter than the controls.

**Figure 5.**
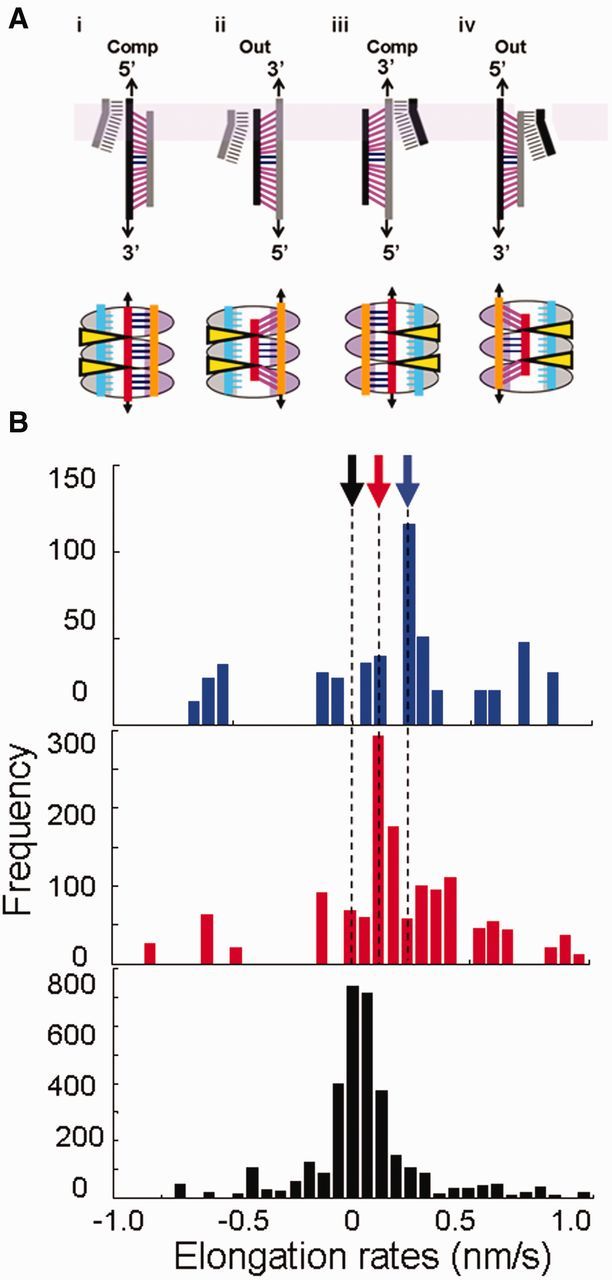
Strand-exchange experiments in the presence of homolog 1000-nt ssDNA bound to Rad51 and full lambda phage dsDNA constructs. (**A**) Schematic representation of the effect of force applied to different ends of the dsDNA constructs during strand-exchange experiments. The dsDNA is pulled from 3′5′ ends with stressed and unstressed base pairs shown in magenta and blue, respectively. Filaments were obtained from PCR fragments amplified using lambda phage as template and labeled with one phosphorylated primer. The strand containing the phosphorylated end was degraded by lambda exonuclease; each type of filament is shown in gray or black. In the representation of strand exchange in the first row, the Rad51 molecules were omitted for better clarity. The lavender band indicates the GC-rich end in lambda phage dsDNA. The ellipses in the second row indicate Rad51 monomers with site I and site II shown in grey and purple, respectively, and the yellow triangles indicate regions occupied by the L1 and L2 loops. The outgoing, complementary, and incoming strands are shown in orange, red and cyan, respectively. The effect of pulling the 3′5′ ends of the complementary strand and 3′5′ ends of the outgoing strand is represented. (**B**) Elongation rate histograms for strand exchange in 1 mM CaCl_2_ and 2 mM ATP while pulling the 3′5′ ends of the outgoing strand (blue), 3′5′ ends of the complementary strand (red) and controls in the presence of ssDNA fragments only (black); the black arrows correspond to molecules that were followed but showed no change in extension and the blue and red arrows indicate characteristic rates of 0.21 and 0.12 nm/s for pulling the outgoing and complementary strands, respectively.

Finally, we performed experiments with Rad51-ssDNA filaments that are homologous to lambda phage dsDNA. In this case, the binding of the dsDNA to the filaments is stable even in the absence of external force. Thus, we allowed the homology searching process to start in the absence of force by incubating the sample at 37°C for 2 min before we applied an external force to the dsDNA. Unlike experiments with non-homologous dsDNA, when the dsDNA is homologous the strand that is complementary to the ssDNA in the Rad51-ssDNA filament is distinguishable from the other dsDNA strand. Figure 5A illustrates the four different experiments we performed using two different lambda phage constructs and two different filaments to show that our results were independent of the filament and/or the construct used in each case. Thus, Figure 5Ai and iii correspond to filaments that bind to the complementary strand which is the strand that is pulled. Figure 5Aii and iv correspond to strand-exchange measurements where the outgoing strands in each construct are pulled. Thus each particular dsDNA backbone strand played the role of either the complementary or the outgoing strand depending on the filament with which it interacted. These results are due to functional differences between the complementary and outgoing strands and not due to sequence differences between the two backbones. When we apply forces between 20 and 35 pN to the 3′5′ ends of the complementary strand, we measure the change in extension with time, which is proportional to the strand-exchange rate (9). The measured slopes are converted into histograms, which are fit to determine the strand-exchange rate, as illustrated in Figure 5B. Analysis of the histograms suggests that when force is applied to the 3′5′ ends of the complementary strand, the strand-exchange rate is smaller than the rate that is observed when the same force is applied to the 3′5′ ends of the outgoing strand. The black arrow indicates the peak corresponding to zero increase in extension obtained with a negative control, whereas the blue and red arrows indicate the strand-exchange rates of 0.21 and 0.12 nm/s for pulling on the outgoing or complementary strands, respectively. In sum, pulling on the complementary strand decreases the observed strand-exchange rate, whereas pulling on the outgoing strand does not.

## DISCUSSION

The experiments that measured the increase in dsDNA extension as a function of time in the presence of non-homologous Rad51-ssDNA filaments suggest that pulling on the 3′5′ ends of one dsDNA strand allows non-homologous dsDNA to bind to and extend along Rad51-ssDNA filaments. No extension is observed when the applied force is <40 pN. For applied forces of ∼53 pN, the observed extension significantly exceeds the controls. For applied forces between 54 and 58 pN, extension versus time curves show pause points that are proportional to the lengths of the ssDNA in the Rad51-ssDNA filaments, suggesting that the dsDNA extends along the entire length of the filament. Furthermore, the pulling on the 3′5′ ends did not simply overcome a barrier to binding. The force is required to maintain the extension, since removing the force resulted in the unbinding of dsDNA that had bound to dsDNA pulled from the 3′5′ ends, as shown in SI. In contrast, applying a force <56 pN to the 3′3′ or 5′5′ ends did not result in extension that is significantly in excess of the extension for the controls.

Earlier results from experiments using RecA-ssDNA filaments showed that pulling on the 3′5′ ends allowed non-homologous dsDNA to substantially extend along RecA-ssDNA filaments (10). Those experiments also showed the applied force was required to stabilize the extension of the non-homologous dsDNA, and that the extension versus time curves showed pause points at positions that were proportional to the length of the ssDNA in the RecA-ssDNA filament. Given the known crystal structure of the RecA-DNA filaments, pulling on the 3′5′ ends of the dsDNA was assumed to decrease the differential extension between the complementary and the outgoing strands. That decrease in differential extension in turn reduces the stress on the base pairs that bind the complementary strand to the filament due to the Watson–Crick pairing between the outgoing and complementary strands. The reduction in stress was assumed to decrease the free energy cost of binding additional dsDNA triplets, which resulted in the substantial extension of non-homologous dsDNA along RecA-ssDNA filaments. In the absence of an external force, earlier experimental results have suggested that the initial interaction between non-homologous dsDNA and RecA-ssDNA filaments is confined to 9–12 bp (11). Thus, the experimental results for Rad51-ssDNA are similar to the previous RecA results.

Similarly, recent studies of the RecA system have suggested that the tension on the base pairs resulting from the differential extension not only limits the number of bases that can bind to site II in the absence of an external force applied to the 3′5′ ends (10), but that the same tension on the bases also drives strand exchange forward (9). It was suggested that the tension on the bases in the strand exchanged conformation where the complementary strand bases are paired with the incoming strand bases is lower than the tension in the initial bound conformation where the complementary strand bases are paired with the outgoing strand bases. This difference in tension arises because the incoming strand backbone is much closer to the helical axis than the outgoing strand backbone. In the RecA system, this proposition was tested by measuring the strand-exchange rate for sequence matched dsDNA when a force of ∼30 pN was applied to the complementary strand in the dsDNA. Those experiments showed a decrease in the strand-exchange rate when the force was applied to the complementary strand. In contrast, the strand-exchange rate did not change when the same force was applied to the outgoing strand. Those results suggested that the differential extension between the complementary strand and its pairing partners results in tension in the bases that drives strand exchange. The RecA results are similar to the experimental results reported here for Rad51-ssDNA filaments.

In sum, the similarity between the Rad51 results and the RecA results, suggests that the differential tension between the complementary strand and its pairing partners limits the number of bases that can bind in site II and drives strand exchange forward.

## SUPPLEMENTARY DATA

Supplementary Data are available at NAR Online.

Supplementary Data
