# Supplementary material for: The differential extension in dsDNA bound to Rad51 filaments may play important roles in homology recognition and strand exchange
Source: Nucleic Acids Res. 2013 Sep 30;42(1):526–33. doi: 10.1093/nar/gkt867 (PMC3874182; doi:10.1093/nar/gkt867)
Supplement: Supplementary Data [file supp_42_1_526__index.html]

The differential extension in dsDNA bound to Rad51 filaments may play important roles in homology recognition and strand exchange — The differential extension in dsDNA bound to Rad51 filaments may play important roles in homology recognition and strand exchange — Supplementary Data 

# The differential extension in dsDNA bound to Rad51 filaments may play important roles in homology recognition and strand exchange

## Supplementary Data

files

**Files in this Data Supplement:**

- Supplementary Data - doc file
