# Supplementary material for: The differential extension in dsDNA bound to Rad51 filaments may play important roles in homology recognition and strand exchange
Source: Nucleic Acids Res. 2013 Sep 30;42(1):526–33. doi: 10.1093/nar/gkt867 (PMC3874182; doi:10.1093/nar/gkt867)
Supplement: Supplementary Data [file supp_gkt867_nar-00846-h-2013-File007.doc]

**Supplementary information**

**The differential extension in dsDNA bound to Rad51 filaments may play important roles in homology recognition and strand exchange**

Claudia Danilowicz^1^, Alexandra Peacock-Villada^1^, Julea Vlassakis^1^, Adrien Facon^1^, Efraim Feinstein^1^, Nancy Kleckner^2^, and Mara Prentiss^1^

^1^Department of Physics, Harvard University, Cambridge, Massachusetts 02138, USA.

^2^Department of Molecular and Cellular Biology, Harvard University, Cambridge, Massachusetts 02138, USA.

Corresponding author: Mara Prentiss

[prentiss@fas.harvard.edu](mailto:prentiss@fas.harvard.edu)

This document contains Supplementary methods, results, and Supplementary Figures S1, S2, S3, S4, and S5.

**Supplementary Methods**

**Preparation of ssDNA samples for strand exchange experiments.**

Fragments were prepared by PCR reactions using lambda phage dsDNA as template. The products were purified using a Macherey-Nagel kit and verified by gel electrophoresis in 0.8 % agarose and TBE buffer with final staining in ethidium bromide. One of the primers was phosphorylated in the 5' end position while the other primer contained a label (fluorescein for the strand exchange in vitro experiments or digoxigenin for the experiments with polystyrene beads). The dsDNA sample was subsequently incubated with lambda exonuclease at 37°C for 15’ in order to hydrolyze the strand that contained the 5' phosphorylated end and generate ssDNA (fluorescein or digoxigenin labeled). The incubation time for this reaction was previously evaluated by incubating a 1 kb fluorescein labeled dsDNA fragment with lambda exonuclease during several time periods, stopped by inactivating the enzyme for 15’ at 75°C, and followed by gel electrophoresis (Supplementary Figure S1A).

**Strand exchange reaction in vitro.**

The ssDNA obtained after the exonuclease reaction was further purified using a Qiagen kit for ssDNA isolation and purification. The concentration was checked by UV absorbance. An aliquot of the ssDNA sample (7.8 μM in nucleotides) was mixed with Rad51 buffer (70 mM Tris-HCl, 2 mM CaCl_2_, 1 mM dithiothreitol, and 50 mM NaCl pH 7.6) containing 2 mM ATP, 2.6 μM human Rad51 (Abcam), and 0.5 μM SSB (single-stranded binding protein) (Epicentre) and incubated at 37°C for 10 minutes. The ratio of ssDNA:Rad51 was calculated in order to achieve a 3:1 ratio, expressed in nucleotides per Rad51 molecule (each Rad51 binds to three bases). For the strand exchange reaction an aliquot of dsDNA (1 kb or full lambda phage) was added, and the reaction was further incubated at 37°C for 90 minutes. Finally the reaction was stopped by adding SDS (final concentration 1%) and Proteinase K (New England Biolabs) (final concentration 1 mg/ml) and incubating at 37°C for 10 minutes. The deproteinized reaction mixture was further analyzed by electrophoresis in 0.8 % agarose gel in TBE buffer. BioRad FX Pro scanner and Quantity One software were used to visualize and quantify the bands (Supplementary Figure S1B and C).

**Stand exchange reaction in the presence of polystyrene and magnetic beads.**

5 kb dsDNA homolog to lambda phage dsDNA containing a digoxigenin label and phosphorylated at the opposite ends was incubated with antidigoxigenin coated polystyrene beads (1.0 μm in diameter). After incubation in the presence of lambda exonuclease at 37°C for one hour, the beads were centrifuged and the buffer was exchanged by Rad51 buffer containing 2 mM ATP, 1 μM human Rad51 (Abcam), and 0.2 μM SSB (single-stranded binding protein) (Epicentre). The sample was incubated at 37°C for 10 minutes, and subsequently lambda phage dsDNA biotinylated at one end was added and incubated for another hour in the presence of 4.5-μm magnetic beads coated with Extravidin. A 5-μl aliquot of this reaction was mixed with 45-μl of glycerol and placed in a glass microcapillary (Supplementary Figure S1D).

**Supplementary Results**

**Demonstration that the Rad51-ssDNA filaments are competent for strand exchange**

The gel assays shown in Supplementary Figure S1A indicate that exonuclease successfully transforms dsDNA into ssDNA. In our strand exchange reactions, the Rad51-ssDNA filaments are formed by combining the ssDNA created by that reaction with free Rad51 in the correct stoichiometric ratios. The resulting Rad51-ssDNA filaments were then combined with homologous dsDNA to create strand exchange products. We performed the strand exchange reaction using a variety of ssDNA and dsDNA lengths. In the gel assays, the ssDNA was fluorescently labeled at one end, so it can be readily detected.

Supplementary Figure S1B shows the fluorescence signal due to the label on a 400-nt ssDNA. In the control experiments shown in lane A, the fluorescent ssDNA exhibits a strong band at the expected position of 400 nt ssDNA. In contrast, the results of the strand exchange reaction are shown in lane B. Those results do not show any fluorescent signal at the position characteristic of the 400 nt ssDNA; however, they show a fairly strong band at the expected position of the strand exchange product. This result indicates that the fluorescent label is stable after strand exchange and the subsequent deprotenization. The absence of fluorescence at the original position of the 400 nt ssDNA indicates that the vast majority of the ssDNA participated in the formation of a strand exchange product.

Supplementary Figure S1C shows an experiment that is identical to Figure S1B, except that both the ssDNA and dsDNA were longer. In Supplementary Figure S1C, the ssDNA contains 1000 nts and the dsDNA is the complete sequence of lambda phage, which has ~ 50kbp. Lanes A and B are duplicates of controls samples containing 1000-nt fluorescently labeled ssDNA, 5kb dsDNA (unlabeled and added before loading the wells as an internal standard for quantifications), and lambda phage dsDNA (unlabeled). Both lanes show a strong fluorescence due to the labeled ssDNA, where the fluorescence intensity is quite reproducible. The weak signal appearing for the dsDNA markers is due to residual dye in our electrophoresis set up. In contrast, lane C corresponds to the product of the strand exchange reaction between the Rad51-ssDNA filaments and lambda phage dsDNA. As in Figure S1B lane B, there is negligible fluorescence in the position associated with the ssDNA, indicating that the Rad51-ssDNA filaments participated in the strand exchange reaction that yielded a product appearing in a different position in the gel. For these longer DNAs, the strand exchange product does not form a clear band, possibly because of diffuse propagation through the gel. Given that Figure S1B showed that the fluorescence of the ssDNA is preserved not only in the Rad51-ssDNA filaments, but also in the strand exchange product, the strong reproducible fluorescence signal corresponding to the position of the 1000 nt ssDNA in Figure S1C lanes A and B along with the near elimination of this signal in lane C suggests that almost all of the ssDNA formed the strand exchange product.

Additional support for the assertion that strand exchange does take place even when the ssDNA and dsDNA molecules are long is shown by an assay performed with Rad51-5000 nt ssDNA filaments homolog to lambda phage dsDNA molecules that yielded stable strand exchange products (Supplementary Figure S1D). The Rad51- ssDNA filaments were bound to 1 micron polystyrene beads. The filaments interacted with homolog lambda phage dsDNA molecules attached to a magnetic bead to one end of each dsDNA.. Applying a magnetic force to an aliquot of the sample mixed with glycerol induced the dsDNA to move toward the magnet. As a result of the drag in the liquid, the dsDNA extended as it moved. In each frame, the cyan arrow indicates the position of the 1-μm polystyrene bead attached to the Rad51-ssDNA filament, and the white arrow indicates the position of the magnetic bead attached to the dsDNA. The sequence of these frames shows that as the strand exchange product is dragged across the screen by the magnetic force acting on the magnetic bead, the distances between the fixed features on the surface of the slide change between frames; however, the relative position of the two beads does not change. Given that the 1-μm polystyrene beads are not magnetic, the constant separation of the two beads indicates that the polystyrene bead is attached to the magnetic bead due to the formation of a strand exchange product.


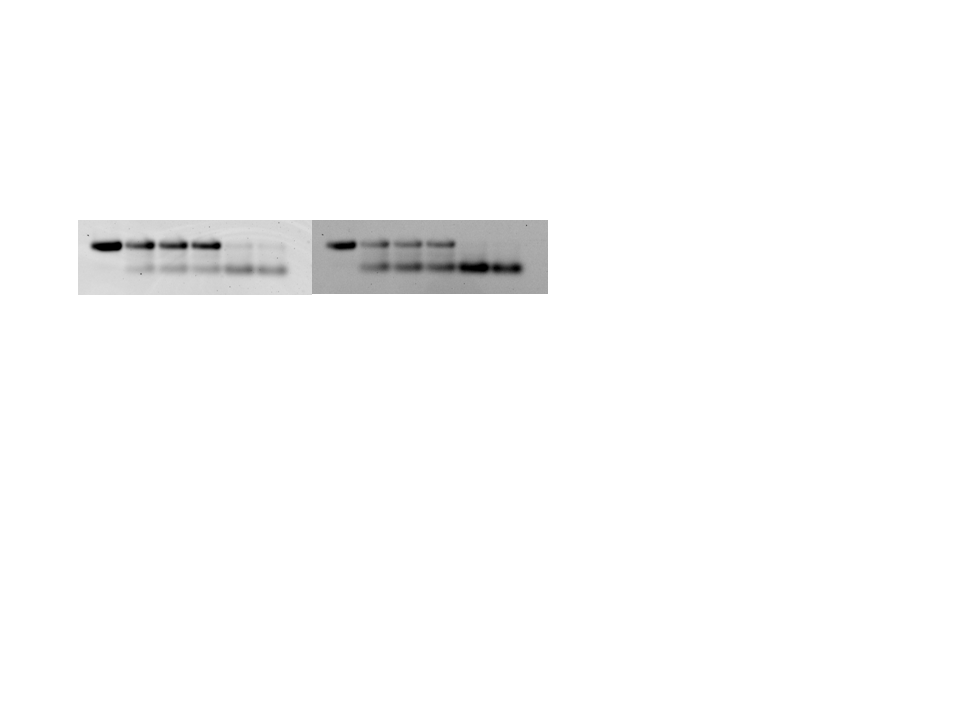


A


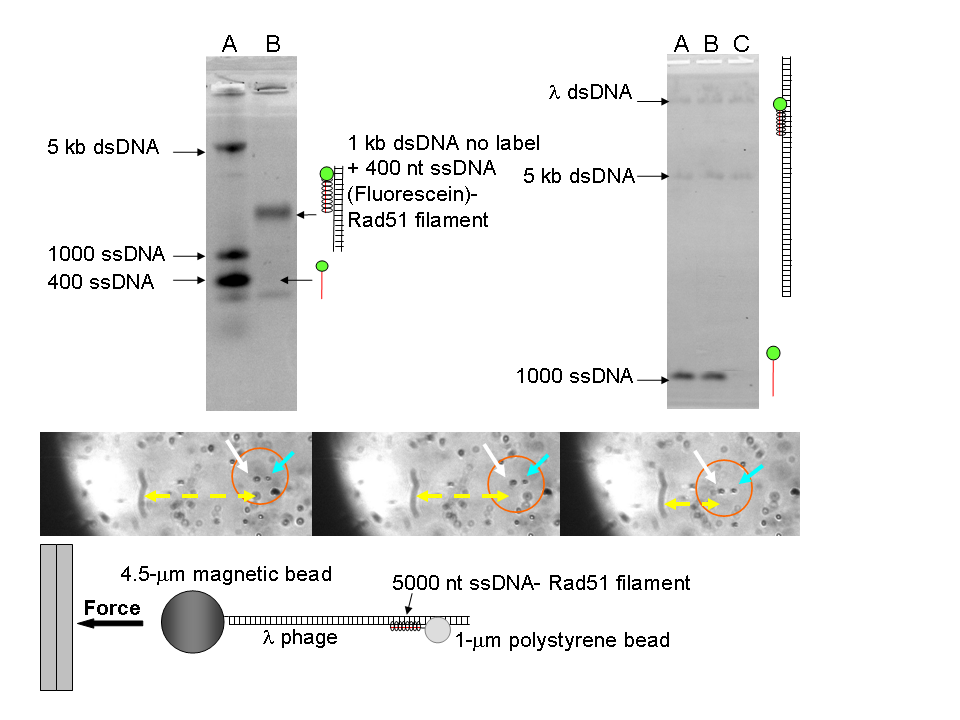


B

C

D

**Supplementary Figure S1** (A) Evolution of the lambda exonuclease reaction by incubating a 1kb fluorescein labeled dsDNA PCR fragment with lambda exonuclease at 37°C for 1, 3, 5,10, and 30 minutes. The reaction was stopped by inactivating the enzyme for 15’ at 75°C and followed by gel electrophoresis and staining in ethidium bromide. The slower band corresponds to unmodified dsDNA (first lane is time 0). Left side: UV detection with BioRad FX Pro gel scanner with ethidium signal becoming dominant and more intense for dsDNA whereas the weaker signal corresponds to ssDNA. Right side: 488 nm excitation showing intense emission for both bands due to the presence of the fluorescein label. A BioRad FX Pro scanner with external 488-nm laser was used. (B) Electrophoresis gel of the strand exchange product with Rad51- 400 ssDNA filaments and 1 kb dsDNA showing labeled ssDNA and dsDNA as markers in lane A and the product of the strand exchange reaction in lane B. (C) Electrophoresis gel of the strand exchange product with Rad51- 1000 ssDNA filaments and lambda phage dsDNA where lanes A and B are the same and correspond to the 1 kb ssDNA obtained after the exonuclease reaction and additional unlabeled markers (5 kb dsDNA and lambda phage dsDNA) and lane C is the product of the strand exchange reaction. (D) Strand exchange in vitro was performed using a Rad51- 5000 nt ssDNA filament homolog to lambda phage and attached to polystyrene beads (cyan arrows) while lambda phage was biotinylated at one end and attached to a magnetic bead (white arrows). An aliquot of the reaction was mixed with glycerol to make the movement of the magnetic beads towards the magnet slow, and images were taken while the magnetic bead dragged the non magnetic bead after strand exchange took place.


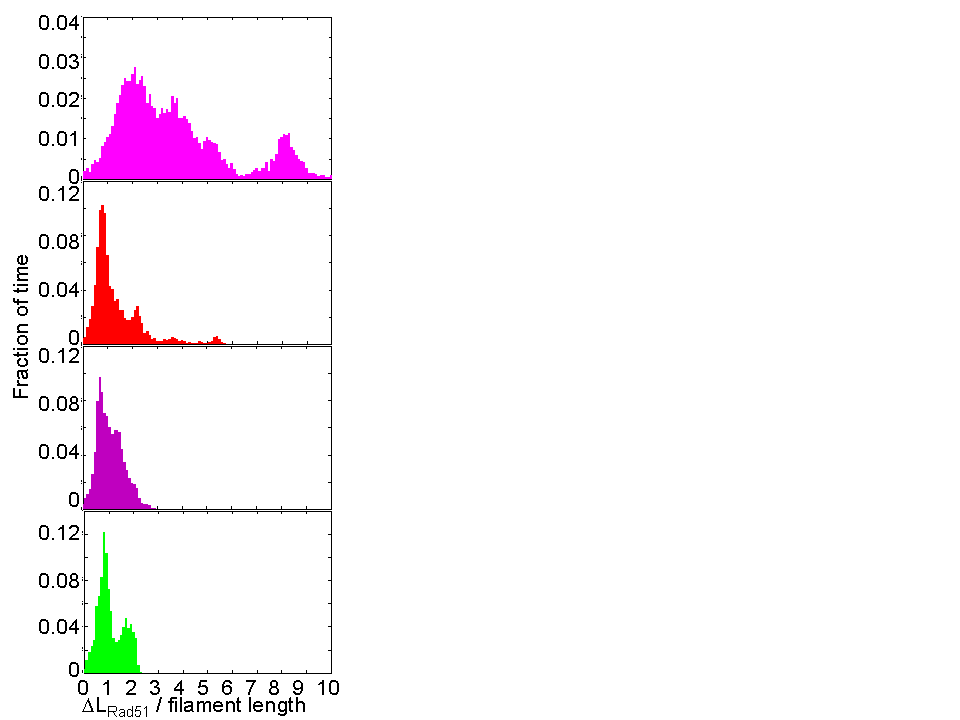


**Supplementary Figure S2** ΔL_Rad51_ probability distributions for periods of ≥ 2sec for all filaments of 400 nts (magenta), 700 nts (red), 1000 nts (purple), and 1600 nts (green) at 54-58 pN.


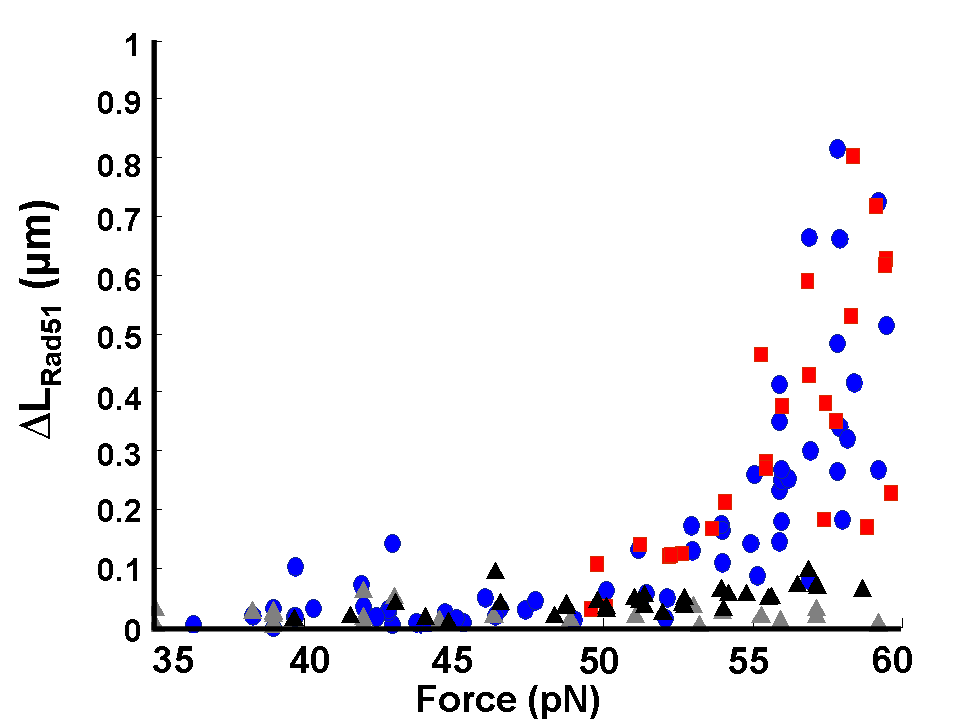


**Supplementary Figure S3** Changes in extension of lambda DNA pulled 3'5' in the presence of Rad51-1000-nt ssDNA from pcDNA3 filaments (blue) and Rad51- circular ssDNA M13mp18 filaments (red) after 120 seconds at constant force (range of forces between 35 and 60 pN). The control extensions for experiments with 1000-nt ssDNA from pcDNA3 and circular ssDNA from M13mp18 and in the absence of protein are shown in gray and black, respectively.


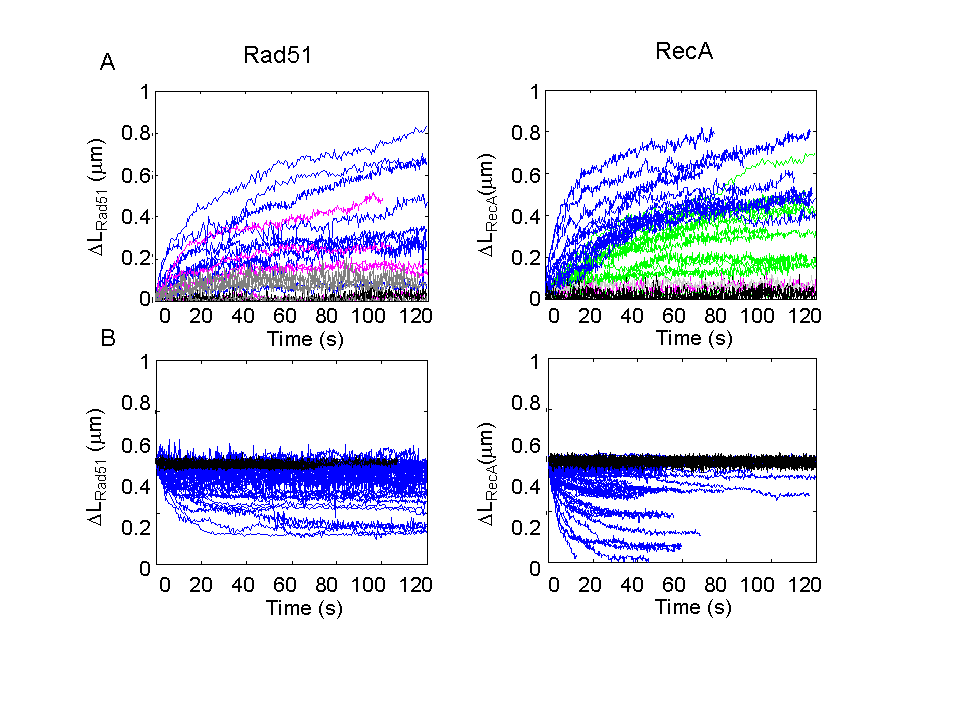


**Supplementary Figure S4** (A) Extension versus time binding profiles for dsDNA pulled 3'5' (blue), 3'3' (green), and 5'5' (magenta) between 56 and 58 pN in the presence of Rad51-ssDNA filaments or RecA-ssDNA filaments. Filaments are 1000-nt long obtained from pcDNA3 non-homolog to lambda dsDNA and incubated with either Rad51 or RecA proteins. (B) Unbinding curves from forces between 50-58 pN after decreasing force to ~40 pN in the presence of Rad51-ssDNA filaments or RecA-ssDNA filaments.


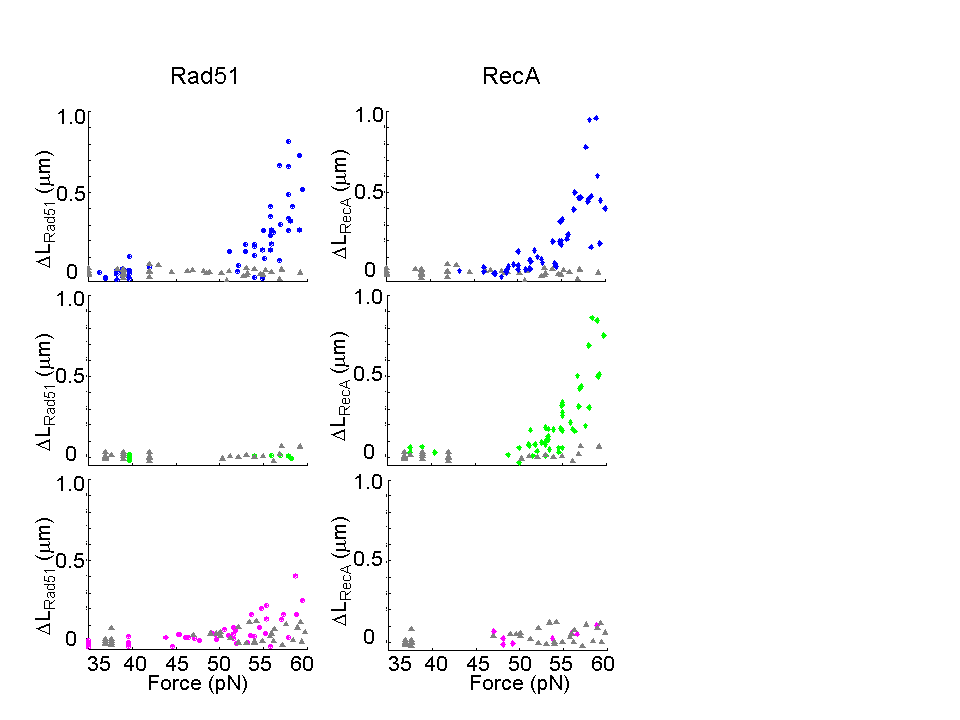


**Supplementary Figure S5** Comparison between different dsDNA pulling modes in the presence of non-homolog Rad51-ssDNA filaments or RecA-ssDNA filaments. Changes in extension of lambda DNA pulled 3'5' (blue), 3'3' (green), and 5'5' (magenta) after 120 seconds at constant force are shown (range of forces between 35 and 60 pN). The control extensions for experiments with 1000-nt ssDNA fragments from pcDNA3 and no protein are shown in gray.
